# Comment on Rohrscheib *et al*. 2016 "Intensity of mutualism breakdown is determined by temperature not amplification of *Wolbachia* genes"

**DOI:** 10.1371/journal.ppat.1006540

**Published:** 2017-09-11

**Authors:** Ewa Chrostek, Luis Teixeira

**Affiliations:** 1 Max Planck Institute for Infection Biology, Berlin, Germany; 2 Instituto Gulbenkian de Ciência, Oeiras, Portugal; 3 Faculdade de Medicina da Universidade de Lisboa, Portugal; Stanford University, UNITED STATES

Rohrscheib *et al*. (PLOS Pathogens, 2016) [1] discuss the interaction between the pathogenicity of the *w*Mel variant *w*MelPop, temperature and Octomom copy number. The effect of temperature on *w*MelPop pathogenicity was already reported in the original work on *w*MelPop [2]. The absence of pathogenicity at low temperatures was also shown before [3]. We have recently demonstrated, in Chrostek and Teixeira 2015, that Octomom copy number determines *w*MelPop pathogenicity [4].

Rohrscheib *et al*. claim to provide evidence that our main conclusions were wrong. We disagree and our reasoning is outlined in the points below.

1) In the first set of experiments Rohrscheib *et al*. show that *w*MelPop pathogenicity in *Drosophila melanogaster* varies with temperature, being stronger at higher temperatures. This confirms previous results [2,3], which were acknowledged and applied in our work (Fig. 3E and Fig. S5H of Chrostek and Teixeira [4]). The authors also show that *Wolbachia* titers vary with temperature, being higher at higher temperature. Finally, Rohrscheib *et al*. also analyze Octomom copy numbers in flies kept at different temperatures. Based on a combination of these analyses the authors conclude that *w*MelPop pathology or levels are not dependent on Octomom copy number.

We disagree with the argument presented to justify the approach outlined above to study the interaction between Octomom copy number and *w*MelPop pathology: “(…), if the Octomom copy number determines *Wolbachia* density, and consequently pathology, we would expect to observe a decrease in Octomom copy number as the extrinsic temperature decreases”. Temperature interacting with pathogenicity does not imply that temperature influences a genetic determinant of pathogenicity—Octomom copy number. Environment can influence phenotype without affecting the genotype, as is the case in many examples of phenotypic plasticity. The setup presented by Rohrscheib *et al*. does not test the influence of Octomom copy number on *w*MelPop pathogenicity correctly. Temperature is a confounding factor already known to influence both *Drosophila* lifespan and *w*MelPop pathogenicity. To directly test the effect of one variable (Octomom copy number) over a phenotype (pathogenicity or *Wolbachia* levels), the variable should be manipulated and tested under stable environmental conditions in which the pathogenicity is known to be expressed. Using this approach we have shown that Octomom copy number determines *w*MelPop pathogenicity at two different temperatures (25°C and 29°C).

2) Rohrscheib *et al*. show that Octomom copy numbers change throughout life of the host. We have previously shown that Octomom copy number is highly variable between flies. In particular, single flies from a non-controlled *w*MelPop *Drosophila* stock have Octomom copy numbers ranging from two to ten copies (Fig. 1A of Chrostek and Teixeira [4]). This variability seems to decrease under selection for Octomom copy number (Fig. 2 and Fig. S2 of Chrostek and Teixeira [4]). However, some variation is either maintained or constantly generated, as selection for Octomom copy number could be reversed either by direct reverse selection or by relaxing selection in high Octomom copy number stocks (Fig. S6 and Fig. S8 of Chrostek and Teixeira [4]).

Rohrscheib *et al*. report substantial changes in Octomom copy number throughout *Drosophila* life. The average Octomom copy number increases more than two fold in the first eight days of adult life and subsequently decreases more than four fold. These large directional changes could be explained by heterogeneity in *w*MelPop Octomom copy number between flies. According to our model, during early adult life of *Drosophila*, *w*MelPop with higher Octomom copy numbers proliferate more than *w*MelPop with lower Octomom copy numbers. Consequently, over time their contribution to the pool of total *w*MelPop in the host population increases and total mean Octomom copy number increases. Next, flies carrying *w*MelPop with high Octomom copy numbers die faster and, therefore, at later time points these *Wolbachia* are depleted from the pool of total *w*MelPop and mean Octomom copy number decreases. This and similar explanations, based on conclusions of Chrostek and Teixeira [4], are discussed and dismissed by Rohrscheib *et al*. However, when we simulate Octomom copy number variation in pooled samples from a heterogeneous population, we can observe initial increase of mean Octomom copy number followed by a later decrease (Fig 1 in this text), similar to the dynamics reported by Rohrscheib *et al*.

**Fig 1:**
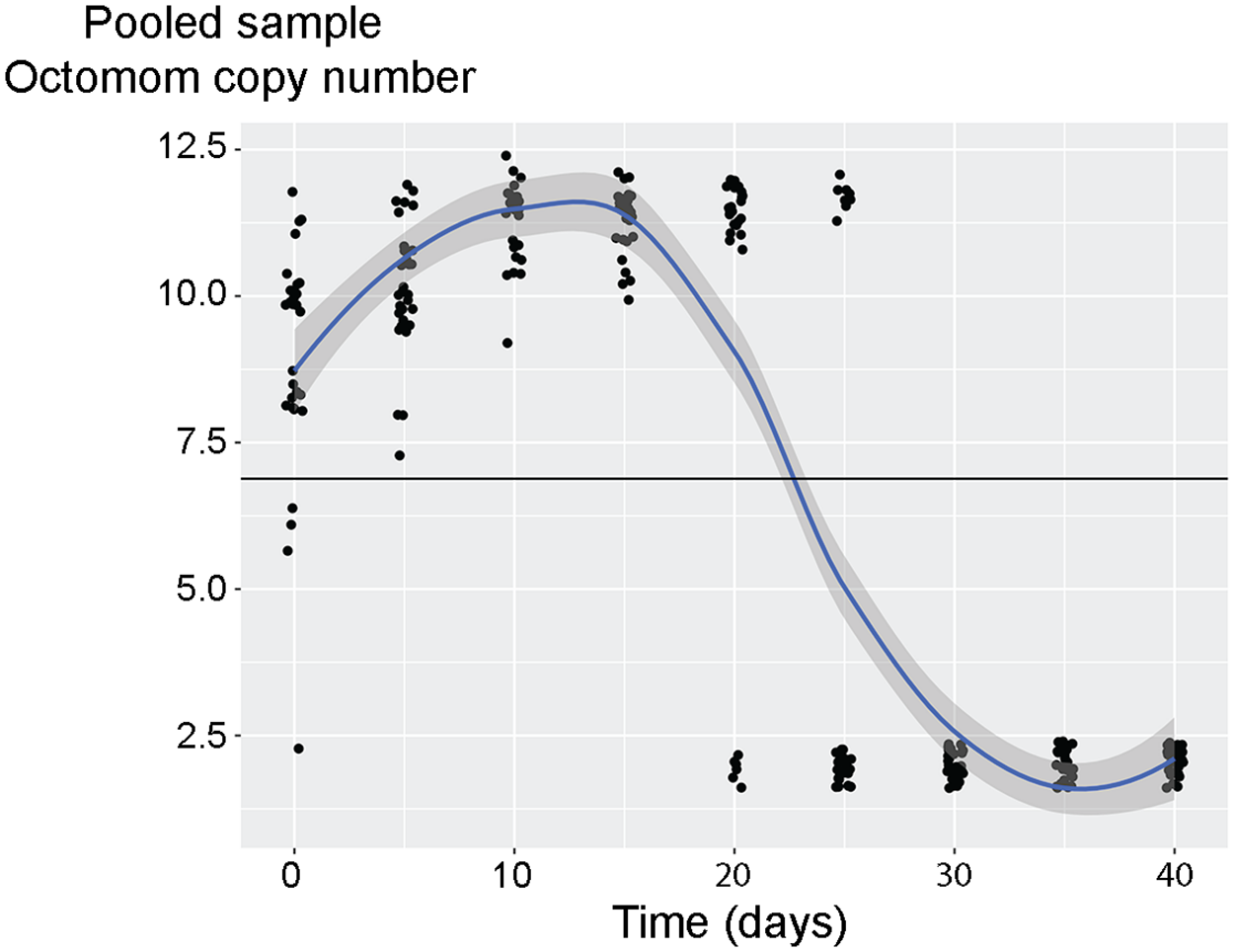
Simulation of Octomom copy number variation over time for 30 samples of five flies from a heterogeneous population. This simulation is based on an initial population constituted of 50% flies carrying *w*MelPop with two Octomom copies and 50% flies carrying *w*MelPop with twelve Octomom copies. *Wolbachia* levels data used for this simulation are published as Fig. 3C (Dataset S6) and survival data as Fig. S5G (Dataset S4) in [4]. Both datasets include flies raised and kept at 25°C. The script (S1 Text) and data (S1 and S2 Datasets) for the simulation are provided as supplementary data. The black line indicates the average Octomom copy number of all the simulated samples. The smooth conditional mean is included for illustrative purposes.

3) The most puzzling result presented in Rohrscheib *et al*. is that average Octomom copy number equals 1 to 1.5 at various temperatures and it is about 0.5 copy at 18°C. These estimates are much lower than estimates from our previous publications [4,5] and from an independent report on *w*MelPop in *Drosophila melanogaster* [6] co-authored by some of the authors of the paper under discussion. *w*MelPop strains with zero copies of Octomom were only reported in *Aedes aegypti* cells and mosquitoes (*w*MelPop-CLA and wMelPop-PGYP) [6].

This striking difference in *w*MelPop Octomom copy numbers estimates between this and previous reports requires a more detailed characterization and validation.

1. None of the graphs or tables with Octomom qPCR data shows control *w*Mel variants with only one Octomom copy in the genome. Presenting the data for these lines is necessary to validate and interpret qPCR results.
2. The calculation of Octomom copy number is crucial for this discussion. It is not clear how the authors calculate specific Octomom copy numbers based on the qPCR results for *WD0550* and *WD0508*. To determine Octomom copy numbers per genome the authors state that they used the delta-delta Ct method [7], but cite the paper on the Pfaffl method [8]. These are similar but not equivalent methods. The Pfaffl method requires determination of the efficiencies of the primers and includes them in the formula, while the delta-delta Ct assumes that the efficiencies are equal to 2 (100%). Nonetheless, both methods use a calibrator sample and the calculated fold changes are expressed relative to this calibrator [7,8]. The use of any of these two methods to calculate gene copy number variation requires a calibrator sample with known ratio of gene of interest to the reference gene [9]. In this particular case a calibrator with known Octomom copy number is required. Otherwise, both methods produce adimensional values, appropriate to compare the magnitude of change between samples within a single experiment, but inappropriate to estimate Octomom copy number. Since Rohrscheib *et al*. did not specify what they used as a calibrator, it is impossible to determine what the presented qPCR results represent.
3. Finally, sequencing of the *w*MelPop used in this study would show if it is otherwise identical to the *Drosophila melanogaster w*MelPop sequenced in [5] and [6] or if it acquired other mutations, similarly to the *w*MelPop-CLA and wMelPop-PGYP in *Aedes aegypti* cells and mosquitoes [6].

4) When calculating *Wolbachia* levels in *Drosophila* the authors also report *Wolbachia* to host genome estimates: “reaching an average maximum density of approximately 181.6 *Wolbachia* genomes to 1 *Drosophila* genome.” It is not explained how these estimates are calculated. Again, to calculate *Wolbachia* to host genomes ratios using the Pfaffl or delta-delta Ct methods a calibrator sample with known *Wolbachia* to host genomes ratio should have been used, which is not mentioned in the paper.

5) The authors characterize a new pathogenic *w*Mel strain, determine that it has low Octomom copy numbers, and use this as an argument for the Octomom copy number being unrelated to pathology. Postulating that amplification of Octomom causes *w*MelPop pathogenicity does not imply that all pathogenic *w*Mel variants or *Wolbachia* strains have Octomom amplification. Therefore, this result is irrelevant for the discussion on *w*MelPop pathogenicity.

6) Finally, in the discussion the authors are not accurate in the description of our previous experiments. They state that in our setup we selected for *w*MelPop pathogenesis: “While both selected for increased or decreased *w*MelPop pathology for a minimum of 14 generations, based on high/low Octomom copy number [22] or on survival [30],…”, and imply that therefore the phenotypes we observed are due to genetic differences in the host. We did not select for increased or decreased *w*MelPop pathology. We selected strictly for Octomom copy number. Therefore, we controlled this number before determining pathology, hence our claim of causality. The assays were performed at several generations, starting at generation 3 (S2 Table in Chrostek and Teixeira [4]). Based on this protocol, it would be improbable to select for host mutations that, both, change host lifespan and correlate with the Octomom copy number—to the point that selection for one Octomom copy number completely reverts the phenotype. Moreover, we performed the selection experiment twice, in two different host genetic backgrounds, which renders the selection on the host even more unlikely.

In summary:

1. Octomom copy number determining pathogenicity is compatible with the previously known fact that temperature influences *w*MelPop pathogenicity.
2. That both factors determine pathogenicity does not imply that they influence each other.
3. The variation in Octomom copy numbers over time in samples from non-controlled *w*MelPop populations can be predicted based on phenotypes associated with different Octomom copy numbers.
4. The low Octomom copy numbers in the *w*MelPop flies reported in Rohrscheib *et al*. are very different from the results reported before. These data require better validation and characterization.

None of the experiments performed in Rohrscheib *et al*. directly tests the influence of Octomom copy number on pathogenicity and growth of *Wolbachia*. There was no attempt to replicate any of the experiments presented in Chrostek and Teixeira [4]. Overall, experiments and conclusions of Chrostek and Teixeira hold and the evidence corroborates the causal relationship between Octomom amplification and wMelPop virulence.

## Materials and methods

### Simulation of Octomom copy number variation over time in pools of flies from a heterogeneous population

The data used for this simulation were previously published in [4]. *Wolbachia* levels data were published as Fig. 3C (Dataset S6) and survival data as Fig. S5G (Dataset S4). All flies used in these experiments were raised and kept at 25°C. In this simulation the Octomom copy number of *w*MelPop is assumed to be fixed throughout time and equal to two or twelve. The initial simulated population of flies was composed of 50% flies carrying *w*MelPop with two Octomom copies per genome and 50% flies carrying *w*MelPop with twelve Octomom copies. The proportion of these flies in the population at different time points was calculated from the survival data. Proportion of flies carrying *w*MelPop with two Octomom copies in the population at a given time point is equal to percentage of these flies surviving at that time point divided by the sum of the percentages of survival of flies carrying wMelPop with either Octomom copy number. Proportion of flies carrying *w*MelPop with twelve Octomom copies is equal to one minus the proportion of flies carrying *w*MelPop with two copies of Octomom. Thirty samples of five flies per time point were sampled in the simulation. The odds of sampling a fly carrying two or twelve copies of Octomom, at each time point, correspond to the proportion of flies carrying *w*MelPop with either Octomom copy number.

The levels of *Wolbachia* in flies carrying *w*MelPop with either Octomom copy number, at a given time point, was calculated from the statistics of the linear regression analysis of *Wolbachia* levels. For each *w*MelPop set a log-linear regression was performed using lm in R. The estimated intercepts and slopes were used to calculate levels of *Wolbachia* at each time point.

For each sample of five flies the Octomom copy number estimate is equal to the sum of the products of *Wolbachia* levels times Octomom copy number for each fly, divided by the sum of the *Wolbachia* levels estimates for each fly.

The script to simulate these samples was written in R [10].

Due to experimental variation the survival of flies carrying *w*MelPop with two Octomom copies was slightly lower that flies carrying *w*MelPop with twelve copies during the first days of the assay. To test the influence of this variation in the outcome of the simulation, survival data can be manually edited to ensure higher (100%) survival of flies carrying *w*MelPop with two Octomom copies in the initial time points of the simulation. This change does not affect the clear initial increase in average Octomom copy number with time. A suggestion on how to perform this alteration is embedded in the script as a comment.

## Supporting information

S1 Text Script to simulate Octomom copy number variation over time in a mixed population. (R)

S1 Dataset Survival data of flies carrying *w*MelPop with two or twelve Octomom copies. (CSV)

S2 Dataset *Wolbachia* levels in flies carrying *w*MelPop with two or twelve Octomom copies. (CSV)
